# Control and inhibition analysis of complex formation processes

**DOI:** 10.1186/1742-4682-9-33

**Published:** 2012-08-03

**Authors:** Takashi Saitou, Keiko Itano, Daisuke Hoshino, Naohiko Koshikawa, Motoharu Seiki, Kazuhisa Ichikawa, Takashi Suzuki

**Affiliations:** 1 Division of Mathematical Science, Department of Systems Innovation, Graduate School of Engineering Science, Osaka University, 1-3 Machikaneyama, Toyonaka, Osaka, 560-8581, Japan; 2 Division of Cancer Cell Research, Institute of Medical Science, University of Tokyo, 4-6-1 Shirokanedai, Minato-ku, Tokyo, 108-8639, Japan; 3 Mathematical Oncology, Institute of Medical Science, University of Tokyo, 4-6-1 Shirokanedai, Minato-ku, Tokyo, 108-8639, Japan

**Keywords:** Control analysis, Complex formation, Biochemical reaction kinetics, Proteinase inhibitors, Cancer invasion, Matrix metalloproteinases

## Abstract

**Background:**

Proteolytic degradation of the extracellular matrix (ECM) is a key event in tumour metastasis and invasion. Matrix metalloproteinases (MMPs) are a family of endopeptidases that degrade most of the components of the ECM. Several broad-spectrum MMP inhibitors (MMPIs) have been developed, but have had little success due to side effects. Thus, it is important to develop mathematical methods to provide new drug treatment strategies. Matrix metalloproteinase 2 (MMP2) activation occurs via a mechanism involving complex formation that consists of membrane type 1 MMP (MT1-MMP), tissue inhibitor of matrix metalloproteinase 2 (TIMP2) and MMP2. Here, we focus on developing a method for analysing the complex formation process.

**Results:**

We used control analysis to investigate inhibitor responses in complex formation processes. The essence of the analysis is to define the response coefficient which measures the inhibitory efficiency, a small fractional change of concentration of a targeting molecule in response to a small fractional change of concentration of an inhibitor. First, by using the response coefficient, we investigated models for general classes of complex formation processes: chain reaction systems composed of ordered steps, and chain reaction systems and site-binding reaction systems composed of unordered multi-branched steps. By analysing the ordered step models, we showed that parameter-independent inequalities between the response coefficients held. For the unordered multi-branched step models, we showed that independence of the response coefficients with respect to equilibrium constants held. As an application of our analysis, we discuss a mathematical model for the MMP2 activation process. By putting the experimentally derived parameter values into the model, we were able to conclude that the TIMP2 and MMP2 interaction is the most efficient interaction to consider in selecting inhibitors.

**Conclusions:**

Our result identifies a new drug target in the process of the MMP2 activation. Thus, our analysis will provide new insight into the design of more efficient drug strategies for cancer treatment.

## Background

Metastasis and invasion are a major cause of death in cancer patients, and thus preventing this secondary spread of the tumour is an important aspect of cancer therapy. A prerequisite for the migration of endothelial cells through the extracellular matrix (ECM) is the initiation of a biochemical pathway responsible for the proteolytic degradation of these structural barriers. Matrix metalloproteinases (MMPs) are a family of endopeptidases that degrade most of the components of the ECM [1,2]. Several broad-spectrum MMP inhibitors (MMPIs) have been developed, some of which have been used in clinical trials for cancer treatment [3]. However, these MMP inhibitors have had little success due to side effects, implying that these clearly lacked selectivity in their action. Most MMPs are inhibited by MMPIs that bind to the active sites of MMPs. Similarities in active sites of MMPs pose obstacles to the design of specific inhibitors. It is thus important to find alternatives to these approaches to increase specificity. Therefore, it is worth developing mathematical methods in analysing biochemical reaction pathways to provide new drug treatment strategies.

Matrix metalloproteinase 2 (MMP2) was proposed as a potential therapeutic target, based on its high-level expression in many human tumours and its ability to degrade type IV collagen [4]. The activation of MMP2 proenzyme is processed by membrane type 1 matrix metalloproteinase (MT1-MMP) [5-7]. Under physiologic conditions, MMP2 is secreted as a latent form, pro-MMP2, and it has been established that its activation occurs via a mechanism involving a complex formation that consists of MT1-MMP, tissue inhibitor of matrix metalloproteinase 2 (TIMP2) and pro-MMP2. Here, focusing on the MMP2 activation process, we develop a mathematical method which quantifies a response of systems to an inhibitor and classifies interactions in order of the inhibitory efficiency. The goal of this study is to identify the most efficient interaction to consider in selecting inhibitors.

In order to achieve this goal, we use control analysis, a method of sensitivity analysis which is widely used in many fields (see for example [8]). Methods of obtaining quantitative measures of control in metabolic pathways were developed by Kacser and Burns [9] and Heinrich and Rapoport [10,11] (for a review, see [12]). The essence of the analysis is to define the response coefficient which measures the inhibitory efficiency, a small fractional change of concentration of a targeting molecule in response to a small fractional change of concentration of an inhibitor. First, using the response coefficient, we investigate models for general classes of complex formation processes: chain reaction systems composed of ordered steps, and chain reaction systems and site-binding reaction systems composed of unordered multi-branched steps. By analysing the ordered step models, we show that parameter-independent inequalities between response coefficients hold. In the unordered multi-branched step models, we assume there are no cooperative reactions, i.e. the equilibrium constant for binding a site of molecule B to a site of another molecule A is independent of whether any of the other sites of molecule A are occupied. This assumption satisfies the detailed balance condition, but it puts a stronger constraint on the system (the importance of the detailed balance condition in kinetic modelling was discussed in [13-15]). Under this assumption, we show that independence of the response coefficients with respect to the equilibrium constants holds. These results indicate that the inhibitory efficiency depends on the topology of pathway networks. As an application of our analysis, we investigate a mathematical model for the MMP2 activation process [16]. In the model, MT1-MMP is dimerized, bound to TIMP2 and forms a quadruple complex by binding proMMP2, MT1-MT1T2M2, which contributes to activation of proMMP2. We try to identify the most efficient interaction to consider in selecting inhibitors by putting the experimentally derived parameter values into the model.

Therefore, our method can serve as a tool for quantifying the inhibitory efficiency and allows us to determine the most efficient method of selecting the inhibitor in complex formation processes. Application of the method to the MMP model may provide a new tool for designing more efficient drug strategies for cancer treatment.

## Results

### The response coefficient as a measure of the inhibitory efficiency

Here, we explain fundamentals of control analysis. The key point of the analysis is to introduce the response coefficient, which describes how a variable such as molecular concentration responds to variation of parameters such as rate constants. In this paper, we would like to consider the response of an inhibitor concentration. Mathematically, the response is expressed as

$$\begin{array}{l} R=\lim_{\delta [I]\to 0}\frac{\delta F/F}{\delta [I]/[I]}\\ \;=\frac{\partial F}{\partial [I]}\frac{\left[I\right]}{F}=\frac{\partial \ln F}{\partial \ln[I]}\text{,} \end{array}$$

where *F* denotes the objective function, which will be taken as a molecular concentration in the steady state of the system. Therefore, the response coefficient *R* represents a fractional change of the objective function *F*, *δF*/*F*, in response to a fractional change in an inhibitor concentration, *δ*[*I*]/[*I*], in the limit as *δ*[*I*] tends to zero. Introducing an inhibitor requires construction of a new model which consists of the original species and a new inhibitor. Instead of introducing new inhibitors, we here consider the change of the objective function in response to changes in the equilibrium constants. To see why this alternative method is valid, let us consider a simple Michaelis–Menten type enzymatic reaction

$$\text{E}+\text{S}\begin{array}{c} \to \\ \leftarrow \end{array}\text{ES}\to E+P\text{.}$$

Suppression of concentration [*ES*] leads to suppression of P production. Hence, to make analysis simpler, we consider a simpler reaction scheme

$$\text{E}+\text{S}\begin{array}{c} \to \\ \leftarrow \end{array}\text{ES}\text{.}$$

Disturbance of this reaction by an inhibitor in a competitive way is expressed as

$$\begin{array}{l} \text{E}+\text{S}\begin{array}{c} \to \\ \leftarrow \end{array}\text{ES}\\ +\\ \text{I ,}\\ ↑↓\\ \text{EI} \end{array}$$

The steady state solution of this reaction system can be written as

$$\begin{array}{l} {}[ES]=\frac{\left[S\right]}{\alpha K+[S]}{\left[E\right]}_{T},\\ {}[E]+[EI]=\frac{\alpha K}{\alpha K+[S]}{\left[E\right]}_{T\text{,}} \end{array}$$

where [*E*]_*T*_ is the total concentration of the enzyme. The concentration [*ES*] is the enzyme concentration which contributes to P production, while the concentration [*E*] + [*EI*] is the enzyme concentration which does not contribute to P production. The parameter *K*, defined by *K* = *kd*/*ka*, is the equilibrium constant of the enzyme and substrate binding reaction, where *kd* is the dissociation rate constant and *ka* is the association rate constant. The coefficient *α* is written as $\alpha =1+\frac{\left[I\right]}{K_{I}}$, where *K*_*I*_ is the equilibrium constant of the enzyme and inhibitor binding reaction. Because *α* is always greater than 1, an addition of the inhibitor is equivalent to an increase of the equilibrium constant *K*. Therefore, in this paper, instead of adding inhibitors, we perform an inhibition analysis by changing equilibrium constants.

Let us take a look at the basic properties of the response coefficient for a simple model. The response coefficient for *F* with respect to *K* is written as

(1) $$R=\frac{\partial F}{F}/\frac{\partial K}{K}=\frac{\partial \ln F}{\partial \ln K}\text{.}$$

At the steady state, *F* is a general function of total concentrations of molecules and equilibrium constants, and thus we can write *F* = *F*([*E*]_*T*_, [*S*]_*T*_, *K*). Let us see how the response coefficient behaves as the value of the equilibrium constant changes. First, suppose that the total concentration of the substrate is much greater than that of the enzyme, i.e. [*E*]_*T*_ < < [*S*]_*T*_. In this case, the system can be reduced to a linear system. The steady state solution is written simply as

$$[ES]=\frac{{\left[S\right]}_{T}}{K+{\left[S\right]}_{T}}{\left[E\right]}_{T}\text{.}$$

The response coefficient for [*ES*] with respect to *K* is calculated as

$$R^{\mathrm{ES}}=\frac{\partial \ln[ES]}{\partial \ln K}=-\frac{K}{K+{\left[S\right]}_{T}}\text{.}$$

**Figure 1:**
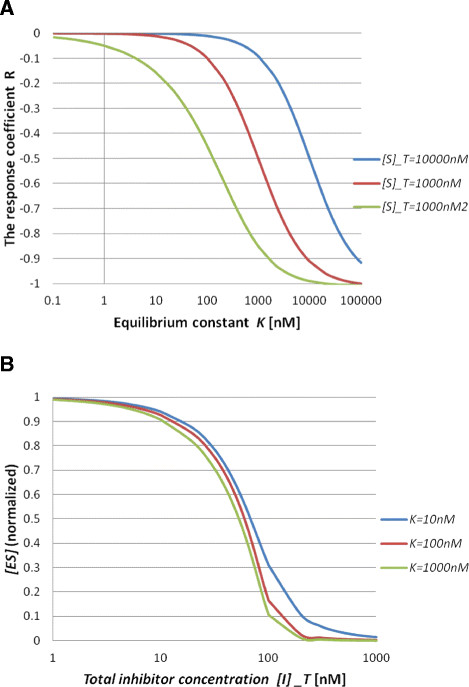
**Behaviour of the steady state for a simple model.** (**A**) Response coefficient *R* as a function of the equilibrium constant *K* for total substrate concentrations [*S*]_*T*_ = 100*nM*, 1000*nM* and 10000*nM*. (**B**) Complex concentration [*ES*] as a function of the total inhibitor concentration [*I*]_*T*_ for equilibrium constant values *K* = 10*nM*, 100*nM* and 1000*nM*.

The minus sign means that a decrease of the complex concentration [*ES*] is caused by an increase of *K*. The response coefficient *R*^*ES*^ goes to 0 as *K* goes to 0, while *R*^*ES*^ goes to −1 as *K* goes to ∞. This indicates that the inhibition becomes more efficient as *K* gets larger. Next, to explore behaviours of the response coefficient in the nonlinear regime (the enzyme concentration is in the same order as the substrate concentration) of the system, we use numerical simulations. We calculate *R*^*ES*^ for three different total substrate concentrations, [*S*]_*T*_ = 1 *nM*, 100 *nM* and 10000 *nM* (Figure 1A). All three curves behave in the same manner in that *R*^*ES*^ goes to 0 as *K* goes to 0, while *R*^*ES*^ goes to −1 as *K* becomes large, as described above. To compare these three curves quantitatively, we introduce the Hill coefficient *n*_*H*_, which quantifies steepness:

$$n_{H}=\frac{\log81}{\log K_{90}/K_{10}}\text{,}$$

where *K*_90_ is the value of *K* such that *R*^*ES*^= − 0.9 and *K*_10_ is the value of *K* such that *R*^*ES*^= − 0.1. We obtain *n*_*H*_ = 1.02 when [*S*]_*T*_ = 10000*nM*, *n*_*H*_ = 0.98 when [*S*]_*T*_ = 100*nM* and *n*_*H*_ = 0.73 when [*S*]_*T*_ = 1*nM*. Thus, the steepness slightly decreases as the total concentration decreases. Furthermore, we calculate the inhibitor concentration dependence of the complex concentration (Figure 1B). Let the value of the total inhibitor concentration necessary to reach a 50% reduction of concentration (*IC*_50_) be a measure of the inhibition. We obtain *IC*_50_ = 67.89*nM* when *K* = 10*nM*, *IC*_50_ = 59.72*nM* when *K* = 100*nM* and *IC*_50_ = 53.20*nM* when *K* = 1000*nM*. We can see that the inhibition becomes more efficient as the equilibrium constant gets larger. Therefore, we conclude that the response coefficient can be a measure of the inhibitory efficiency.

So far, we have discussed behaviours of the response coefficient in a simple model as an example. We would like to generalize this analysis to larger systems which have multiple interactions. From here, we consider models for general classes of complex formation processes: (1) chain reaction systems composed of ordered steps, (2) chain reaction systems composed of unordered multi-branched steps and (3) site-binding reaction systems composed of unordered multi-branched steps.

### Analysis of the ordered step models

We first investigate models for chain reaction systems composed of ordered steps. Let *A*_1_, …, *A*_*n*_ be *n* types of molecules. The reaction scheme of the model is described as

$$\begin{array}{l} A_{1}+A_{2}\begin{array}{c} \overset{k_{1A}}{\to }\\ \underset{k_{1d}}{\leftarrow } \end{array}A_{1}A_{2},\\ A_{1}A_{2}+A_{3}\begin{array}{c} \overset{k_{2A}}{\to }\\ \underset{k_{2d}}{\leftarrow } \end{array}A_{1}A_{2}A_{3},\\ A_{1}A_{2}A_{3}+A_{4}\begin{array}{c} \overset{k_{3A}}{\to }\\ \underset{k_{3d}}{\leftarrow } \end{array}A_{1}A_{2}A_{3}A_{4},\\ \vdots \;\ddots \\ A_{1}\cdots A_{n-1}+A_{n}\begin{array}{c} \overset{k_{n-1A}}{\to }\\ \underset{k_{n-1d}}{\leftarrow } \end{array}A_{1}\cdots A_{n-1}A_{n}\text{.} \end{array}$$

We refer to this model as “Model Sn”. In order to provide a basis for the analysis, we begin with the simple case Model S3, which consists of three molecules: *A*_1_, *A*_2_, *A*_3_. By linear approximation under the assumption [*A*_1_] < < [*A*_2_], [*A*_3_], a simple analytical steady state solution can be derived for molecular concentrations

(2) $$\begin{array}{l} {}[A_{1}]=\frac{{\tilde{K}}_{1}{\tilde{K}}_{2}}{\left({\tilde{K}}_{1}+1\right){\tilde{K}}_{2}+1}{\left[A_{1}\right]}_{T},\\ {}[A_{1}A_{2}]=\frac{{\tilde{K}}_{2}}{\left({\tilde{K}}_{1}+1\right){\tilde{K}}_{2}+1}{\left[A_{1}\right]}_{T},\\ {}[A_{1}A_{2}A_{3}]=\frac{{\tilde{K}}_{2}}{\left({\tilde{K}}_{1}+1\right){\tilde{K}}_{2}+1}{\left[A_{1}\right]}_{T}\text{,} \end{array}$$

where ${\tilde{K}}_{1}=K_{1}/{\left[A_{2}\right]}_{T}$ and ${\tilde{K}}_{2}=K_{2}/{\left[A_{3}\right]}_{T}$. The response coefficients for the complex concentrations with respect to *K*_1_ and *K*_2_ are calculated as

(3) $$\begin{array}{l} R_{1}^{123}=\frac{\partial \ln[A_{1}A_{2}A_{3}]}{\partial \ln{\tilde{K}}_{1}}=-\frac{{\tilde{K}}_{2}{\tilde{K}}_{1}}{\left({\tilde{K}}_{1}+1\right){\tilde{K}}_{2}+1},\\ R_{2}^{123}=\frac{\partial \ln[A_{1}A_{2}A_{3}]}{\partial \ln{\tilde{K}}_{2}}=-\frac{{\tilde{K}}_{2}\left({\tilde{K}}_{1}+1\right)}{\left({\tilde{K}}_{1}+1\right){\tilde{K}}_{2}+1},\\ R_{1}^{12}=\frac{\partial \ln[A_{1}A_{2}]}{\partial \ln{\tilde{K}}_{1}}=-\frac{{\tilde{K}}_{2}{\tilde{K}}_{1}}{\left({\tilde{K}}_{1}+1\right){\tilde{K}}_{2}+1},\\ R_{2}^{12}=\frac{\partial \ln[A_{1}A_{2}]}{\partial \ln{\tilde{K}}_{2}}=\frac{1}{\left({\tilde{K}}_{1}+1\right){\tilde{K}}_{2}+1},\\ R_{1}^{1}=\frac{\partial \ln[A_{1}]}{\partial \ln{\tilde{K}}_{1}}=\frac{{\tilde{K}}_{2}+1}{\left({\tilde{K}}_{1}+1\right){\tilde{K}}_{2}+1},\\ R_{2}^{1}=\frac{\partial \ln[A_{1}]}{\partial \ln{\tilde{K}}_{2}}=\frac{1}{\left({\tilde{K}}_{1}+1\right){\tilde{K}}_{2}+1}\text{,} \end{array}$$

where *R*_*i*_^123^, *R*_*i*_^12^ and *R*_*i*_^1^(*i* = 1, 2) denote the response coefficients for [*A*_1_*A*_2_*A*_3_], [*A*_1_*A*_2_] and [*A*_1_] with respect to *K*_*i*_, respectively. As can be seen from (3), the following inequalities between the response coefficients hold: *R*_1_^123^ > *R*_2_^123^, *R*_2_^12^ > *R*_1_^12^ and *R*_1_^1^ > *R*_2_^1^. The inequality *R*_1_^123^ > *R*_2_^123^ shows that inhibitory efficiency of the *A*_3_ binding reaction is larger than that of the *A*_2_binding reaction. The sign of *R*_1_^12^ is negative, while that of *R*_2_^12^ is positive. This means that the complex concentration [*A*_1_*A*_2_] increases by the inhibition of the *A*_2_ binding reaction.

**Figure 2:**
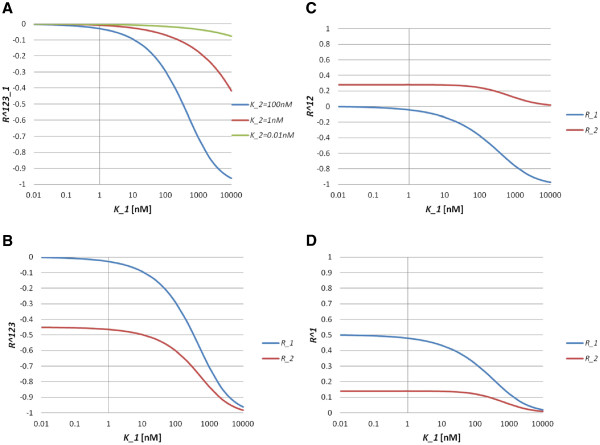
**Behaviours of the response coefficients for Model S3.** (**A**) Response coefficient *R*_1_^123^ as a function of *K*_1_ for *K*_2_ = 100*nM*, 1 *nM* and 0.01 *nM*. (**B**) Response coefficient *R*_*i*_^123^ (*i* = 1, 2) as a function of *K*_1_. Blue and red lines indicate *R*_1_^123^ and *R*_2_^123^, respectively. (**C**) Response coefficient *R*_*i*_^12^(*i* = 1, 2) as a function of *K*_1_. Blue and red lines indicate *R*_1_^12^ and *R*_2_^12^, respectively. (**D**) Response coefficient *R*_*i*_^1^(*i* = 1, 2) as a function of *K*_1_. Blue and red lines indicate *R*_1_^1^ and *R*_2_^1^, respectively. In (B)–(D), we set *K*_2_ = 100*nM*.

In the nonlinear regime of Model S3, we cannot solve for the steady state solution explicitly. Thus, we instead investigate whether the same relations hold by numerical simulations. In the simulations, we set the total concentrations as [*A*_*i*_]_*T*_ = 100*nM*(*i* = 1, 2, 3). From the simulation results, we can see the following: *R*_1_^123^ depends on the values of *K*_1_and *K*_2_ (Figure 2A). The inequality *R*_1_^123^ > *R*_2_^123^ holds for a wide range of *K*_1_ values (Figure 2B). The sign of *R*_1_^12^ is negative, while that of *R*_2_^12^ is positive (Figure 2C). *R*_1_^1^ and *R*_2_^1^ are both positive and satisfy *R*_1_^1^ > *R*_2_^1^ (Figure 2D). Therefore, we have shown that the parameter-independent inequalities hold both in the linear and the nonlinear regimes of Model S3.

To investigate whether similar relations hold in larger systems, we next consider Model S4. To linearize the model, we assume [*A*_1_]_*T*_ < < [*A*_2_]_*T*_, [*A*_3_]_*T*_, [*A*_4_]_*T*_. As in Model S3, the steady state solution can be derived for molecular concentrations

(4) $$\begin{array}{l} {}[A_{1}]=\frac{{\tilde{K}}_{1}{\tilde{K}}_{2}{\tilde{K}}_{3}}{\left(\left({\tilde{K}}_{1}+1\right){\tilde{K}}_{2}+1\right){\tilde{K}}_{3}+1}{\left[A_{1}\right]}_{T},\\ {}[A_{1}A_{2}]=\frac{{\tilde{K}}_{2}{\tilde{K}}_{3}}{\left(\left({\tilde{K}}_{1}+1\right){\tilde{K}}_{2}+1\right){\tilde{K}}_{3}+1}{\left[A_{1}\right]}_{T},\\ {}[A_{1}A_{2}A_{3}]=\frac{{\tilde{K}}_{3}}{\left(\left({\tilde{K}}_{1}+1\right){\tilde{K}}_{2}+1\right){\tilde{K}}_{3}+1}{\left[A_{1}\right]}_{T},\\ {}[A_{1}A_{2}A_{3}A_{4}]=\frac{1}{\left(\left({\tilde{K}}_{1}+1\right){\tilde{K}}_{2}+1\right){\tilde{K}}_{3}+1}{\left[A_{1}\right]}_{T}\text{.} \end{array}$$

**Figure 3:**
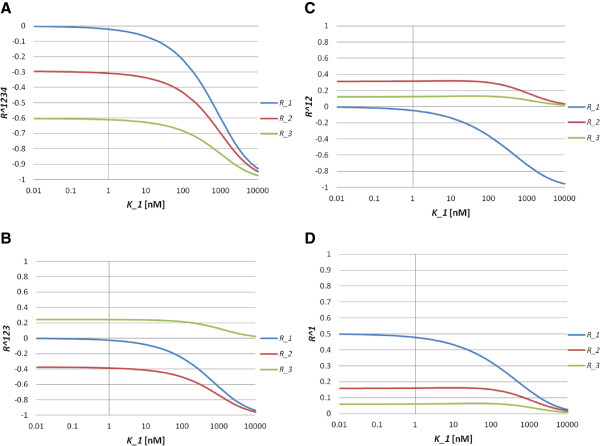
**Behaviours of the response coefficients for Model S4.** (**A**) Response coefficient *R*_*i*_^1234^ (*i* = 1,2,3) as a function of *K*_1_. Blue, red and green lines indicate *R*_1_^1234^, *R*_2_^1234^ and *R*_3_^1234^, respectively. (**B**) Response coefficient *R*_*i*_^123^(*i* = 1, 2, 3) as a function of *K*_1_. Blue, red and green lines indicate *R*_1_^123^, *R*_2_^123^ and *R*_3_^123^, respectively. (**C**) Response coefficient *R*_*i*_^12^(*i* = 1, 2, 3) as a function of *K*_1_. Blue, red and green lines indicate *R*_1_^12^, *R*_2_^12^ and *R*_3_^12^, respectively. (**D**) Response coefficient *R*_*i*_^1^(*i* = 1, 2, 3) as a function of *K*_1_. Blue, red and green lines indicate *R*_1_^1^, *R*_2_^1^ and *R*_3_^1^, respectively. For all, we set *K*_2_ = *K*_3_ = 100*nM*.

The response coefficients are calculated as

(5) $$\begin{array}{l} R_{1}^{1234}=-\frac{{\tilde{K}}_{3}{\tilde{K}}_{2}{\tilde{K}}_{1}}{{\tilde{K}}_{3}\left(\left({\tilde{K}}_{1}+1\right){\tilde{K}}_{2}+1\right)+1}\text{,}\;R_{2}^{1234}=-\frac{{\tilde{K}}_{3}{\tilde{K}}_{2}\left({\tilde{K}}_{1}+1\right)}{{\tilde{K}}_{3}\left(\left({\tilde{K}}_{1}+1\right){\tilde{K}}_{2}+1\right)+1}\text{,}\\ R_{3}^{1234}=-\frac{{\tilde{K}}_{3}\left(\left({\tilde{K}}_{1}+1\right){\tilde{K}}_{2}+1\right)}{{\tilde{K}}_{3}\left(\left({\tilde{K}}_{1}+1\right){\tilde{K}}_{2}+1\right)+1},\;R_{1}^{123}=-\frac{{\tilde{K}}_{3}{\tilde{K}}_{2}{\tilde{K}}_{1}}{{\tilde{K}}_{3}\left(\left({\tilde{K}}_{1}+1\right){\tilde{K}}_{2}+1\right)+1}\text{,}\\ R_{2}^{123}=-\frac{{\tilde{K}}_{3}{\tilde{K}}_{2}\left({\tilde{K}}_{1}+1\right)}{{\tilde{K}}_{3}\left(\left({\tilde{K}}_{1}+1\right){\tilde{K}}_{2}+1\right)+1}\text{,}\;R_{3}^{123}=\frac{1}{{\tilde{K}}_{3}\left(\left({\tilde{K}}_{1}+1\right){\tilde{K}}_{2}+1\right)+1},\\ R_{1}^{12}=-\frac{{\tilde{K}}_{3}{\tilde{K}}_{2}{\tilde{K}}_{1}}{{\tilde{K}}_{3}\left(\left({\tilde{K}}_{1}+1\right){\tilde{K}}_{2}+1\right)+1}\text{,}\;R_{2}^{12}=\frac{{\tilde{K}}_{3}+1}{{\tilde{K}}_{3}\left(\left({\tilde{K}}_{1}+1\right){\tilde{K}}_{2}+1\right)+1}\text{,}\\ R_{3}^{12}=\frac{1}{{\tilde{K}}_{3}\left(\left({\tilde{K}}_{1}+1\right){\tilde{K}}_{2}+1\right)+1},\;R_{1}^{1}=\frac{\left({\tilde{K}}_{2}+1\right){\tilde{K}}_{3}+1}{{\tilde{K}}_{3}\left(\left({\tilde{K}}_{1}+1\right){\tilde{K}}_{2}+1\right)+1}\text{,}\\ R_{2}^{12}=\frac{{\tilde{K}}_{3}+1}{{\tilde{K}}_{3}\left(\left({\tilde{K}}_{1}+1\right){\tilde{K}}_{2}+1\right)+1}\text{,}\;R_{3}^{12}=\frac{1}{{\tilde{K}}_{3}\left(\left({\tilde{K}}_{1}+1\right){\tilde{K}}_{2}+1\right)+1}\text{,} \end{array}$$

where $R_{i}^{1234}=\frac{\partial \ln[A_{1}A_{2}A_{3}A_{4}]}{\partial \ln{\tilde{K}}_{i}}$, $R_{i}^{123}=\frac{\partial \ln[A_{1}A_{2}A_{3}]}{\partial \ln{\tilde{K}}_{i}}$, $R_{i}^{12}=\frac{\partial \ln[A_{1}A_{2}]}{\partial \ln{\tilde{K}}_{i}}$ and $R_{i}^{1}=\frac{\partial \ln[A_{1}]}{\partial \ln{\tilde{K}}_{i}}(i=1,2,3)$. From the above expression (5), we can see that the inequalities *R*_1_^1234^ > *R*_2_^1234^ > *R*_3_^1234^, *R*_3_^123^ > *R*_1_^123^ > *R*_2_^123^, *R*_2_^12^ > *R*_3_^12^ > *R*_1_^12^ and *R*_1_^1^ > *R*_2_^1^ > *R*_3_^1^ hold. In the simulations, we set total concentrations [*A*_*i*_]_*T*_ = 100*nM*(*i* = 1, 2, 3, 4). We can see that the inequalities *R*_1_^1234^ > *R*_2_^1234^ > *R*_3_^1234^, *R*_3_^123^ > *R*_1_^123^ > *R*_2_^123^, *R*_2_^12^ > *R*_3_^12^ > *R*_1_^12^ and *R*_1_^1^ > *R*_2_^1^ > *R*_3_^1^ hold for a wide range of *K*_1_ values (Figure 3). The sign of *R*_1_^123^ is positive, while those of *R*_2_^123^ and *R*_3_^123^ are negative (Figure 3B). *R*_1_^12^ and *R*_2_^12^ are positive, while *R*_3_^12^ is negative (Figure 3C). *R*_1_^1^, *R*_2_^1^ and *R*_3_^1^ are positive (Figure 3D). These results suggest that the inequalities relating the response coefficients represent basic properties of the ordered step models.

We can generalize these results to the *n* chain reaction model: Model Sn. Assuming [*A*_1_]_*T*_ < < [*A*_2_]_*T*_, …, [*A*_*n*_]_*T*_, the steady state solution can be obtained as

(6) $$\begin{array}{l} {}[A_{1}]=\frac{{\tilde{K}}_{n-1}\cdots {\tilde{K}}_{1}}{D}{\left[A_{1}\right]}_{T},\\ \vdots \\ {}[A_{1}\cdots A_{i}]=\frac{{\tilde{K}}_{n-1}\cdots {\tilde{K}}_{i}}{D}{\left[A_{1}\right]}_{T}\\ \vdots \\ {}[A_{1}A_{2}\cdots A_{n}]=\frac{1}{D}{\left[A_{1}\right]}_{T}\text{,} \end{array}$$

where $D=1+{\tilde{K}}_{n-1}\left(1+{\tilde{K}}_{n-2}\left(1+\cdots \left(1+{\tilde{K}}_{1}\right)\right)\right)$. The response coefficients are calculated as

(7) $$\begin{array}{l} R_{n-1}^{1\cdots i}=\frac{1}{D},\\ \vdots \\ R_{i+1}^{1\cdots i}=\frac{1+{\tilde{K}}_{n-1}\left(1+\cdots \left(1+{\tilde{K}}_{i+2}\right)\right)}{D},\\ R_{i}^{1\cdots i}=-\frac{{\tilde{K}}_{n-1}\cdots {\tilde{K}}_{i}\left(1+{\tilde{K}}_{i-1}\left(1+\cdots \left(1+{\tilde{K}}_{1}\right)\right)\right)}{D},\\ \vdots \\ R_{1}^{1\cdots i}=-\frac{{\tilde{K}}_{n-1}\cdots {\tilde{K}}_{1}}{D}\text{.} \end{array}$$

where $R_{i}^{j}=\frac{\partial \ln[A_{1}\cdots A_{j}]}{\partial \ln{\tilde{K}}_{i}}$. Therefore, the inequalities are as follows:

$$\begin{array}{l} R_{1}^{1\cdots n}>R_{2}^{1\cdots n}>\cdots >R_{n-1}^{1\cdots n},\\ \vdots \\ R_{i+1}^{1\cdots i}>R_{i+2}^{1\cdots i}>\cdots >R_{n-1}^{1\cdots i}>R_{1}^{1\cdots i}>\cdots >R_{i}^{1\cdots i},\\ \vdots \\ R_{1}^{1}>R_{2}^{1}>\cdots >R_{n-1}^{1}\text{.} \end{array}$$

Thus, we have shown that the parameter-independent inequalities hold in Model Sn. The inequalities indicate that the reactions can be sorted in order of the inhibitory efficiency, which is independent of the values of the equilibrium constants.

### Analysis of the unordered multi-branched step models – chain reaction systems

Here, we consider chain reaction systems with unordered multi-branched steps. In the unordered multi-branched step models, we assume that there are no cooperative reactions, i.e. the affinity of any reaction is independent of whether other sites are occupied. Let *A*_1_, …, *A*_*n*_ be *n* types of molecules. All possible complexes in the general chain reaction system are listed as follows:

$$\begin{array}{l} A_{1}\;A_{2}\;A_{3}\;\cdots \;A_{n-1}\;A_{n}\\ \;A_{1}A_{2}\;A_{2}A_{3}\;\cdots \;\cdots \;\cdots \;A_{n-1}A_{n}\\ \;A_{1}A_{2}A_{3}\;\cdots \;\cdots \;A_{n-2}A_{n-1}A_{n}\\ \;\ddots \;⋰\\ \;A_{1}A_{2}\cdots A_{n} \end{array}$$

We refer to this model as “Model Cn”. The number of complexes is *N*_*S*_ = *n*(*n* + 1)/2 and the number of reactions is *N*_*I*_ = *n*(*n* − 1)(*n* + 1)/2. As an example, let us first consider the *n* = 3 case. All possible complexes can be listed as

A1A2A3A1A2 A2A3 A1A2A3

**Figure 4:**
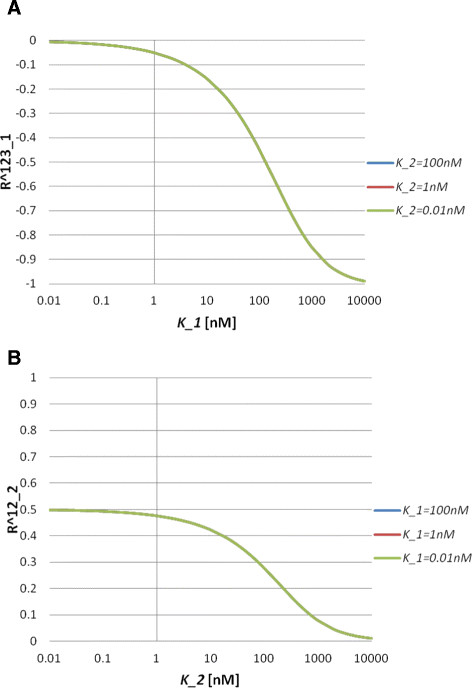
**Behaviours of the response coefficients for Model C3.** (**A**) Response coefficient *R*_1_^123^ as a function of *K*_1_ for *K*_2_ = 100*nM*, 1*nM* and 0.01*nM*. (**B**) Response coefficient *R*_2_^12^ as a function of *K*_2_ for *K*_1_ = 100*nM*, 1*nM* and 0.01*nM*.

In order to deal with the model analytically, we linearize the system. By linear approximation under the assumption [*A*_2_]_*T*_ < < [*A*_1_]_*T*,_[*A*_3_]_*T*_, the steady state solution for molecular concentrations is written simply as

(8) $$\begin{array}{l} {}[A_{2}]=\frac{{\tilde{K}}_{1}{\tilde{K}}_{2}}{\left({\tilde{K}}_{1}+1\right)\left({\tilde{K}}_{2}+1\right)}{\left[A_{2}\right]}_{T},\\ {}[A_{1}A_{2}]=\frac{{\tilde{K}}_{2}}{\left({\tilde{K}}_{1}+1\right)\left({\tilde{K}}_{2}+1\right)}{\left[A_{2}\right]}_{T},\\ {}[A_{2}A_{3}]=\frac{{\tilde{K}}_{1}}{\left({\tilde{K}}_{1}+1\right)\left({\tilde{K}}_{2}+1\right)}{\left[A_{2}\right]}_{T},\\ {}[A_{1}A_{2}A_{3}]=\frac{1}{\left({\tilde{K}}_{1}+1\right)\left({\tilde{K}}_{2}+1\right)}{\left[A_{2}\right]}_{T}\text{,} \end{array}$$

where ${\tilde{K}}_{1}=K_{1}/{\left[A_{1}\right]}_{T}$, ${\tilde{K}}_{2}=K_{2}/{\left[A_{3}\right]}_{T}$. The response coefficients for molecular concentrations with respect to the equilibrium constants are given by

(9) $$\begin{array}{l} R_{1}^{123}=-\frac{{\tilde{K}}_{1}}{{\tilde{K}}_{1}+1}\text{,}\;R_{2}^{123}=-\frac{{\tilde{K}}_{2}}{{\tilde{K}}_{2}+1},\\ R_{1}^{12}=-\frac{{\tilde{K}}_{1}}{{\tilde{K}}_{1}+1}\text{,}\;R_{2}^{12}=\frac{1}{{\tilde{K}}_{2}+1},\\ R_{1}^{23}=\frac{1}{{\tilde{K}}_{1}+1}\text{,}\;R_{2}^{23}=-\frac{{\tilde{K}}_{2}}{{\tilde{K}}_{2}+1},\\ R_{1}^{2}=\frac{1}{{\tilde{K}}_{1}+1}\text{,}\;R_{2}^{2}=\frac{1}{{\tilde{K}}_{2}+1}\text{,} \end{array}$$

where $R_{i}^{123}=\frac{\partial \ln[A_{1}A_{2}A_{3}]}{\partial \ln{\tilde{K}}_{i}}$, $R_{i}^{12}=\frac{\partial \ln[A_{1}A_{2}]}{\partial \ln{\tilde{K}}_{i}}$, $R_{i}^{23}=\frac{\partial \ln[A_{2}A_{3}]}{\partial \ln{\tilde{K}}_{i}}$ and $R_{i}^{2}=\frac{\partial \ln[A_{2}]}{\partial \ln{\tilde{K}}_{i}}(i=1,2)$. An important observation is that the response coefficients with respect to ${\tilde{K}}_{i}$ only depend on${\tilde{K}}_{i}$, i.e. $R_{i}=R_{i}({\tilde{K}}_{i})$. We call this property independence of the response coefficients. This property comes from the assumption that there are no cooperative reactions, because, as shown previously, the ordered step models do not have this property. Numerical simulation results also show independence of the response coefficients in the nonlinear regime of the model (Figure 4). In the simulations, we set [*A*_*i*_]_*T*_ = 100*nM*(*i* = 1, 2, 3). The response coefficient *R*_1_^123^ does not depend on *K*_2_ for a wide range of *K*_1_ values (Figure 4A). The sign of the response coefficient *R*_2_^12^is positive, which means [*A*_1_*A*_2_] increases by the inhibition of *K*_2_ (Figure 4B). The independence of *R*_2_^12^ in terms of *K*_2_also holds for a wide range of *K*_1_ values (Figure 4B). The independence of response coefficients means that inhibitory efficiency is determined by the values of the equilibrium constants.

We next explore Model C4. All possible complexes are listed as

$$\begin{array}{l} A_{1}\;A_{2}\;A_{3}\;A_{4}\\ \;A_{1}A_{2}\;A_{2}A_{3}\;A_{3}A_{4}\\ \;A_{1}A_{2}A_{3}\;A_{2}A_{3}A_{4}\\ \;A_{1}A_{2}A_{3}A_{4} \end{array}$$

**Figure 5:**
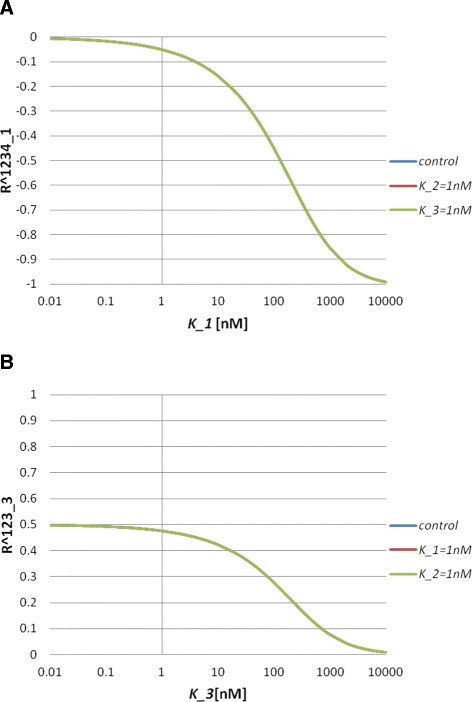
**Behaviours of the response coefficients for Model C4.** (**A**) Response coefficient *R*_1_^1234^ as a function of *K*_1_ for different *K*_2_ and *K*_3_ values. Blue (control) line indicates *K*_2_ = *K*_3_ = 100*nM*, red line indicates *K*_2_ = 1*nM*, *K*_3_ = 100*nM*, and green line indicates *K*_2_ = 100*nM*, *K*_3_ = 1*nM*. (**B**) Response coefficient *R*_3_^123^ as a function of *K*_3_ for different *K*_1_ and *K*_2_ values. Blue (control) line indicates *K*_1_ = *K*_2_ = 100*nM*, red line indicates *K*_1_ = 1*nM*, *K*_2_ = 100*nM*, and green line indicates *K*_1_ = 100*nM*, *K*_2_ = 1*nM*.

We investigate this model only by means of numerical simulations. In the simulations, we set [*A*_*i*_]_*T*_ = 100*nM*(*i* = 1, 2, 3, 4). The response coefficient *R*_1_^1234^ does not depend on *K*_2_ and *K*_3_ for a wide range of *K*_1_ values (Figure 5A). The independence of *R*_3_^123^ in terms of *K*_1_ and *K*_2_ also holds for a wide range of *K*_3_ values (Figure 5B).

### Analysis of the unordered multi-branched step models – site-binding systems

Here, we apply control analysis to the models in which molecule A has *n* binding sites for the binding of *B*_*i*_(*i* = 1, ⋯, *n*). Schematically, all possible complexes are listed as

$$\begin{array}{l} A\;B_{1}\;B_{2}\;\cdots \;B_{n}\\ \;{\text{AB}}_{1}\;{\text{AB}}_{2}\;\cdots \;{\text{AB}}_{n}\\ \;{\text{AB}}_{1}B_{2}\;{\text{AB}}_{1}B_{3}\;\cdots \;{\text{AB}}_{n-1}B_{n}\\ \;\ddots \;\vdots \\ \;{\text{AB}}_{1}\cdots B_{n-1}\;\cdots \;{\text{AB}}_{2}\cdots B_{n}\\ \;{\text{AB}}_{1}\;\cdots \;B_{n} \end{array}$$

**Figure 6:**
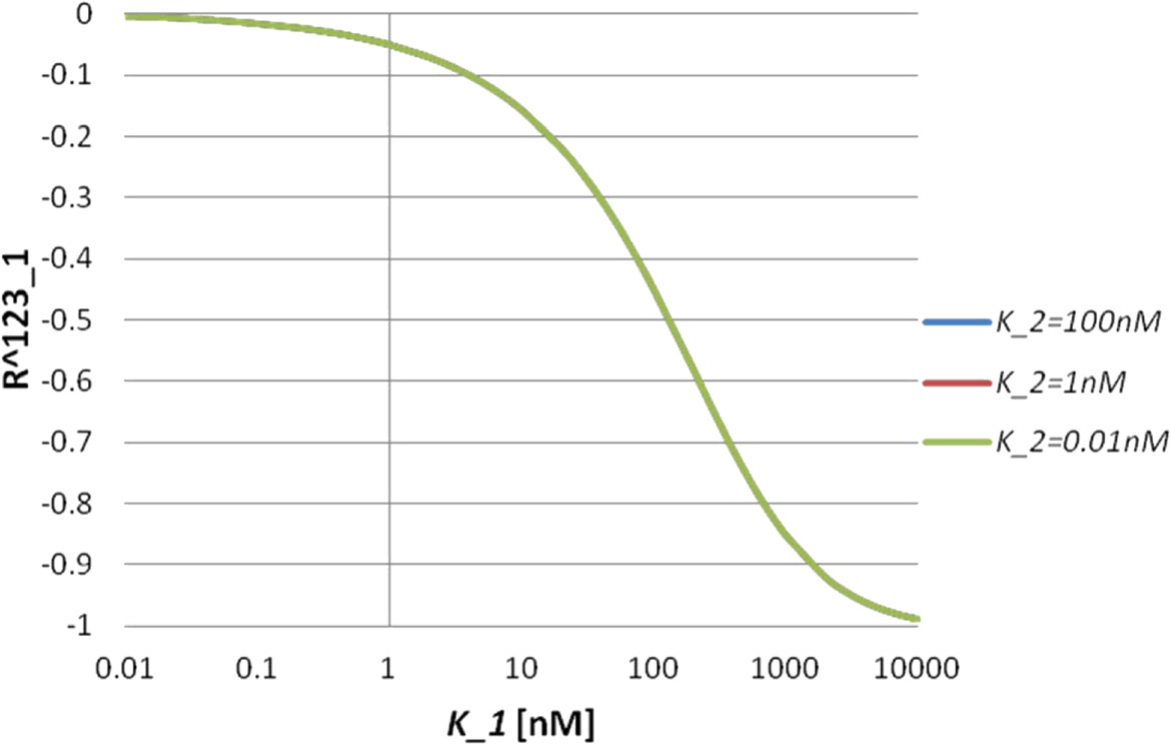
**Behaviour of the response coefficient for Model SB3.** Response coefficient *R*_1_^123^ as a function of *K*_1_ for *K*_2_ = 100*nM*, 1*nM* and 0.01*nM*. Here, we set *K*_3_ = 100*nM*

We refer to this model as “Model SBn”. We assume that each equilibrium constant *K*_*i*_ for binding the site of B_i_to each site of A is independent of whether any of the other sites of A are occupied. By linear approximation under the assumption [*A*]_*T*_ < < [*B*_1_]_*T*_, ⋯, [*B*_*n*_]_*T*_, the steady state solution for molecular concentrations is given by

(10) $$\begin{array}{l} {}[A]=\left(\prod_{j=1}^{n}K_{j}\right)A_{1\cdots n},\\ {}[AB_{i}]=\left(\prod_{j\neq i}^{n}K_{j}\right)A_{1\cdots n}\text{,}\;i=1,\cdots ,n\\ \vdots \\ {}[AB_{i1}\cdots B_{\mathrm{ik}}]=\left(\prod_{j\neq i1,\cdots ,ik}^{n}K_{j}\right)A_{1\cdots n}\text{,}\;i1,\cdots ,ik(i1\neq \cdots \neq ik)=1,\cdots ,n\\ \vdots \\ {}[AB_{1}\cdots B_{n}]=A_{1\cdots n}\text{,} \end{array}$$

where $A_{1\cdots n}=\left(\prod_{j=1}^{n}(1+K_{j})\right)$. The response coefficients for [*AB*_*i*1_⋯*B*_*ik*_] with respect to *K*_*j*_ are given by

(11) $$R_{j}^{i1\cdots ik}=\begin{array}{c} -\frac{{\tilde{K}}_{j}}{1+{\tilde{K}}_{j}}\;if\;j=i1,\cdots ,ik(i1\neq \cdots \neq ik),\\ \frac{1}{1+{\tilde{K}}_{j}}\;if\;j\neq i1,\cdots ,ik(i1\neq \cdots \neq ik)\text{,} \end{array}$$

where $R_{j}^{i1\cdots ik}=\frac{\partial \ln[A_{i1}\cdots A_{\mathrm{ik}}]}{\partial \ln K_{j}}$. Thus, the independence of the response coefficients also holds in this model. We further explore the nonlinear regime of the model by using numerical simulations. We consider Model SB3 as an example. In the simulations, we set [*A*]_*T*_ = [*B*_1_]_*T*_ = ⋯ = [*B*_*n*_]_*T*_ = 100*nM*. We can see that the independence of *R*_1_^123^ holds in a wide range of *K*_1_ (Figure 6). Therefore, together with the results from the chain reaction systems, the independence of the response coefficients is an intrinsic property of unordered multi-branched step models under the assumption that there are no cooperative reactions.

### An application - MT1-MMP/TIMP2/MMP2 complex formation model

As an application of our analysis, we here investigate the MT1-MMP/TIMP2/MMP2 complex formation model [16]. In this model, MT1-MMP is dimerized, bound to TIMP2 and forms a quadruple complex by binding proMMP2, MT1-MT1T2M2, which contributes to activation of proMMP2. The model has all possible pathways that could contribute to the formation and dissociation of complexes. All possible complexes formed by the mentioned biochemical reactions are listed below:

$$\begin{array}{l} \;\text{MT}1\;\text{T}2\;\text{M}2\\ \;\text{MT1-MT}1\;\text{MT1T}2\;\text{T2M}2\\ \;\text{MT1-MT1T}2\;\text{MT1T2M}2\\ \;\text{MT1T2-MT1T}2\;\text{MT1-MT1T2M}2\\ \;\text{MT1T2-MT1T2M}2\\ \;\text{MT1T2M2-MT1T2M}2 \end{array}$$

Rate constants of the reactions are summarized in Table 1. Although a simpler model was proposed previously in [18], we use the model described above. As reported in [16], taking into consideration the transient dynamics of the MMP2 activation, the model we use here is more appropriate than the model proposed in [18].

**Table 1:** Experimentally derived parameter values for the MMP model

**Parameters**	**Values**	**Remarks**
[*MT*1]_*T*_	*100nM*	From Karagiannis 2004 [18]& Hoshino 2012 [16]
[*T*2]_*T*_	*50-100nM*	From Karagiannis 2004 [18] & Hoshino 2012 [16]
[*M*2]_*T*_	*100nM*	From Karagiannis 2004 [18] & Hoshino 2012 [16]
*k*_*a*(*MT*1−*MT*1)_	*2/μM/s*	From Hoshino 2012 [16]
*k*_*d*(*MT*1−*MT*1)_	*0.01/s*	From Hoshino 2012 [16]
*k*_*a*(*MT*1−*T*2)_	*2.74/μM/s*	From Toth 2000 [19]
*k*_*d*(*MT*1−*T*2)_	*0.0001/s*	From Toth 2000 [19]
*k*_*a*(*T*2−*M*2)_	*0.14/μM/s*	From Olson 1997 [20]
*k*_*d*(*T*2−*M*2)_	*0.0047/s*	From Olson 1997 [20]

The parameters *k*_*a*(*MT*1−*MT*1)_ and *k*_*d*(*MT*1−*MT*1)_ are association and dissociation rate constants of the MT1-MMP dimer formation, respectively. The parameters*k*_*a*(*MT*1−*T*2)_ and *k*_*d*(*MT*1−*T*2)_ are association and dissociation rate constants of the MT1-MMP and TIMP2 binding reaction, respectively. The parameters *k*_*a*(*T*2−*M*2)_ and *k*_*d*(*T*2−*M*2)_ are association and dissociation rate constants of the TIMP2 and MMP2 binding reaction, respectively.

**Figure 7:**
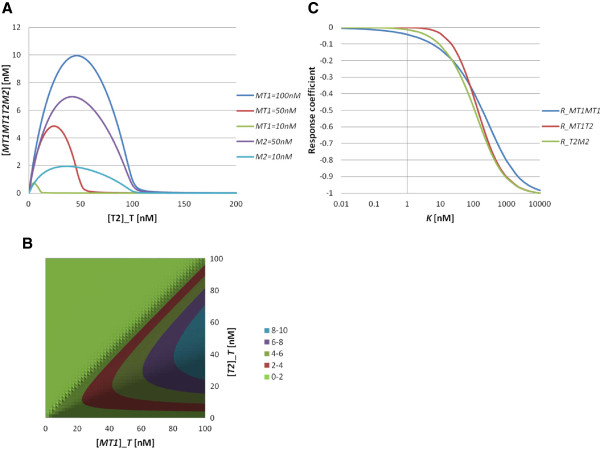
**Behaviour of the steady state of the MMP model.** (**A**) Complex concentration [*MT*1*MT*1*T*2*M*2] as a function of total TIMP2 concentration for different total MT1-MMP and MMP2 concentrations. Blue line indicates [*MT*1]_*T*_ = 100*nM*, [*M*2]_*T*_ = 100*nM*. Red line indicates [*MT*1]_*T*_ = 50*nM*, [*M*2]_*T*_ = 100*nM*. Green line indicates [*MT*1]_*T*_ = 10*nM*, [*M*2]_*T*_ = 100*nM*. Purple line indicates [*MT*1]_*T*_ = 100*nM*, [*M*2]_*T*_ = 50*nM*. Cyan line indicates [*MT*1]_*T*_ = 100*nM*, [*M*2]_*T*_ = 10*nM*. (**B**) Concentration map of the complex concentration [*MT*1*MT*1*T*2*M*2] as a function of total MT1-MMP and TIMP2 concentrations. Here, we set [*M*2]_*T*_ = 100*nM*. (**C**) The response coefficients of the MMP model for the three interactions: the MT1-MMP dimerization, the MT1-MMP and TIMP2 binding, and the TIMP2 and MMP2 binding. The blue line shows the response coefficient *R*_*MT*1*MT*1_ = ∂ ln [*MT*1*MT*1*T*2*M*2]/ ∂ ln *K*_*MT*1*MT*1_ as a function of *K*_*MT*1*MT*1_, the red line shows the response coefficient *R*_*MT*1*T*2_ = ∂ ln [*MT*1*MT*1*T*2*M*2]/ ∂ ln *K*_*MT*1*T*2_ as a function of *K*_*MT*1*T*2_ and the green line shows the response coefficient *R*_*T*2*M*2_ = ∂ ln [*MT*1*MT*1*T*2*M*2]/ ∂ ln *K*_*T*2*M*2_ as a function of *K*_*T*2*M*2_. Here, we set [*MT*1]_*T*_ = 100*nM*, [*T*2]_*T*_ = 50*nM* and [*M*2]_*T*_ = 100*nM*.

Here, we try to identify the most efficient reaction to consider in selecting inhibitors by putting the experimentally derived parameter values into the model. We focus on the behaviour of the concentration of the quadruple complex MT1-MT1T2M2, which is a binding state of a free MT1-MMP and the ternary complex MT1T2M2, because only this complex contributes to activation of proMMP2. The total TIMP2 concentration dependence of the complex MT1-MT1T2M2 is shown (Figure 7A). The concentration of the quadruple complex has the maximum value at a TIMP-2 concentration of 50 nM (Figure 7B control). This property does not depend on *MT*1]_*T*_ and *M*2]_*T*_. This means that the quadruple complex which contributes to the proMMP2 activation is suppressed at high levels of TIMP2 and attains its maximum only at intermediate TIMP2 concentrations [16-18]. A concentration map in the *MT*1]_*T*_ and *T*2]_*T*_ plane is depicted in Figure 7B. These simulation results indicate that the balance between TIMP2 and MT1-MMP expression is a critical determinant of MMP2 activation. The response coefficients for MT1-MT1T2M2 with respect to the equilibrium constants were also calculated (Figure 7C). This model assumes that there are no cooperative reactions, as in the previous examples, and thus the independence of the response coefficients holds. To compare the three curves *R*_*MT*1*MT*1_, *R*_*MT*1*T*2_ and *R*_*T*2*M*2_ quantitatively, we calculate the steepness. The Hill coefficients are *n*_*H*_ = 0.74 for *R*_*MT*1*MT*1_, *n*_*H*_ = 1.15 for *R*_*MT*1*T*2_ and *n*_*H*_ = 0.95 for *R*_*T*2*M*2_. Thus, their steepnesses are slightly different. By substituting experimentally derived values of the equilibrium constant (Table 2) into the response coefficients, we obtain *R*_*MT*1*MT*1_ = − 0.095(*K*_*MT*1*MT*1_ = 5*nM*), *R*_*MT*1*T*2_ = − 0.0024(*K*_*MT*1*T*2_ = 0.548*nM*) and *R*_*T*2*M*2_ = − 0.15(*K*_*T*2*M*2_ = 33.5714*nM*). Therefore, we conclude that the most effective inhibition is the TIMP2 and MMP2 binding interaction.

**Table 2:** Summary of equilibrium constants

*K*_*MT*1−*MT*1_	5nM
*K*_*MT*1−*T*2_	0.548nM
*K*_*T*2−*M*2_	33.5714nM

Several broad-spectrum MMPIs function by strongly chelating the Zn ion that lies in the MMP active site. Similarities in active sites of MMPs pose obstacles to the design of specific inhibitors [3]. Thus, a new approach to the identification of new drug targets is important. Here, focusing on the MMP2 activation process, we were able to determine that the TIMP2-MMP2 is the most effective interaction. It is reported that TIMP2 interacts with MMP2 through the C-terminal domain of the enzyme that is distinct from the active site [21,22]. Therefore, our result identifies a new drug target in the process of MMP2 activation. Development of low molecular weight compounds capable of effectively and specifically inhibiting the TIMP2 and MMP2 binding interaction will be the subject of future research. Our result can be validated using cell culture systems.

## Conclusions

In this paper, our aim is to quantify the response of a system to the addition of inhibitors and to classify their interactions in order of their inhibitory efficiency. In order to analyse the response systematically, we used control analysis. Using the response coefficients, we revealed that the parameter-independent inequalities between the response coefficients hold in the ordered step models. For the unordered multi-branched step models, we showed that independence of the response coefficients with respect to the equilibrium constants holds. These results indicate that the inhibitory efficiency depends on the topology of the pathway networks. We applied our analysis to a complex formation model describing the formation of complexes of MMP2 and MT1-MMP in the presence of TIMP2 [16]. In the complex formation process between these molecules, there are three interactions, i.e. the MT1-MMP dimerization, the MT1-MMP and TIMP2 binding reaction and the TIMP2 and MMP2 binding reaction. We tried to identify the most efficient interaction to consider in selecting the inhibitors by putting the experimentally derived parameter values into the model. The novel finding of the analysis is that the inhibition of the TIMP2 and MMP2 binding interaction is the most efficient method for suppressing the quadruple complex MT1-MT1T2M2, which contributes to the MMP2 activation. This result identifies a new drug target in the process of MMP2 activation.

Our method can also be applied to other models of complex formation processes. However, there are some weaknesses in the analysis presented here. Throughout our treatment, we have considered only the steady state in a well-stirred environment. For the case that the substrate is a non-diffusible molecule such as ECM, the well-stirred assumption is not valid. Thus, in this case, we should consider a three dimensional compartment model in which the extracellular space is divided into small compartments [16,23]. This will allow us to simulate a reaction–diffusion system. Furthermore, we analysed models in closed systems, but intra- and intercellular transport of molecules should play important roles in biochemical reactions occurring in cells. Thus, spatio-temporal dynamics associated with a mechanism such as a positive feedback loop [24] should be considered.

## Methods

### Numerical computation scheme

We employed the fourth-order Runge–Kutta method to solve systems of ordinary differential equations numerically. In all simulations, the time step was taken as *dt* = 0.001 sec and time evolution was performed up to the time *T* = 100000 sec. In the calculation of the response coefficients, the small fractional change of the equilibrium constant *K* was *δK*/*K* = 0.01.

## Competing interests

The authors declare that they have no competing interests.

## Authors’ contributions

TSa carried out the analytical and numerical computations and drafted the manuscript. KIt, DH, NK, MS, KIc and TSu helped to draft the manuscript. All authors read and approved the final manuscript.
